# Neutrophil-Initiated Myocardial Inflammation and Its Modulation by B-Type Natriuretic Peptide: A Potential Therapeutic Target

**DOI:** 10.3390/ijms20010129

**Published:** 2018-12-31

**Authors:** Saifei Liu, Yuliy Y. Chirkov, John D. Horowitz

**Affiliations:** Cardiology/Clinical Pharmacology Unit, Basil Hetzel Institute, Queen Elizabeth Hospital, University of Adelaide, Adelaide 5011, Australia; saifei.liu@adelaide.edu.au (S.L.); yuliy.chirkov@adelaide.edu.au (Y.Y.C.)

**Keywords:** “neutrophil burst”, BNP, heart failure, takotsubo syndrome

## Abstract

Activation of neutrophils is a critically important component of the innate immune response to bacterial and chemical stimuli, and culminates in the “neutrophil burst”, which facilitates neutrophil phagocytosis via the release of superoxide anion radical (O_2_^−^) from NADPH oxidase. Excessive and/or prolonged neutrophil activation results in substantial tissue injury and increases in vascular permeability—resulting in sustained tissue infiltration with neutrophils and monocytes, and persistent vasomotor dysfunction. Cardiovascular examples of such changes include acute and chronic systolic and diastolic heart failure (“heart failure with preserved ejection fraction”), and the catecholamine-induced inflammatory disorder takotsubo syndrome. We have recently demonstrated that B-type natriuretic peptide (BNP), acting via inhibition of activation of neutrophil NADPH oxidase, is an important negative modulator of the “neutrophil burst”, though its effectiveness in limiting tissue injury is partially lost in acute heart failure. The potential therapeutic implications of these findings, regarding the development of new means of treating both acute and chronic cardiac injury states, are discussed.

## 1. Introduction

### 1.1. Neutrophil Activation, Vascular and Myocardial Inflammation

Neutrophils, as a pivotal component of the innate immune system, make fundamental contributions to host defense against invading micro-organisms. The most important aspect of this role is the phagocytic function of neutrophils, consisting of the engulfing of bacteria into lysosomes, within which they are exposed to potentially lethal oxidative stress. The latter is engendered by a transient process usually termed the “neutrophil burst,” whereby activation of the neutrophil isoform of NADPH oxidase results in the production of large quantities of the superoxide (O_2_^−^) anion radical, together with secondary increases in the generation of hydrogen peroxide and hypochlorous acid (HOCl) [1]. This “neutrophil burst” is associated with a transient but marked increase in neutrophil oxygen uptake and utilization (the “respiratory burst”), and is also characterised by cytokine release, the activation of monocytes and macrophages, the increased release of reactive oxygen species (ROS) such as O_2_^−^ into the extracellular space, the formation of neutrophil extracellular traps (NETs), and eventually the apoptosis of neutrophils [2]. NET formation is initiated by the ejection of neutrophil nuclear chromatin, and may be facilitated by the formation of HOCl—via activation of the neutrophil enzyme myeloperoxidase [3]. Termination of the inflammatory process initiated in association with neutrophil activation potentially begins at the time of neutrophil apoptosis, thus leading to what is normally a brief but potent phagocytic response.

The purpose of this review is to shed light on some of the factors recently recognised to modulate the extent of an acute “neutrophil burst”, and also the timing of resolution of this inflammatory response. In doing so, we will focus on the implications of neutrophil activation in acute forms of (non-infective) cardiovascular disease states, and particular forms of impaired left ventricular function. In particular, we will examine growing evidence that B-type natriuretic peptide (BNP)—which is increasingly released from the myocardium in heart failure and myocardial inflammatory states—is a substantial modulator of neutrophil activation and associated ROS production, as well as the potential therapeutic significance of this discovery.

**Figure 1:**
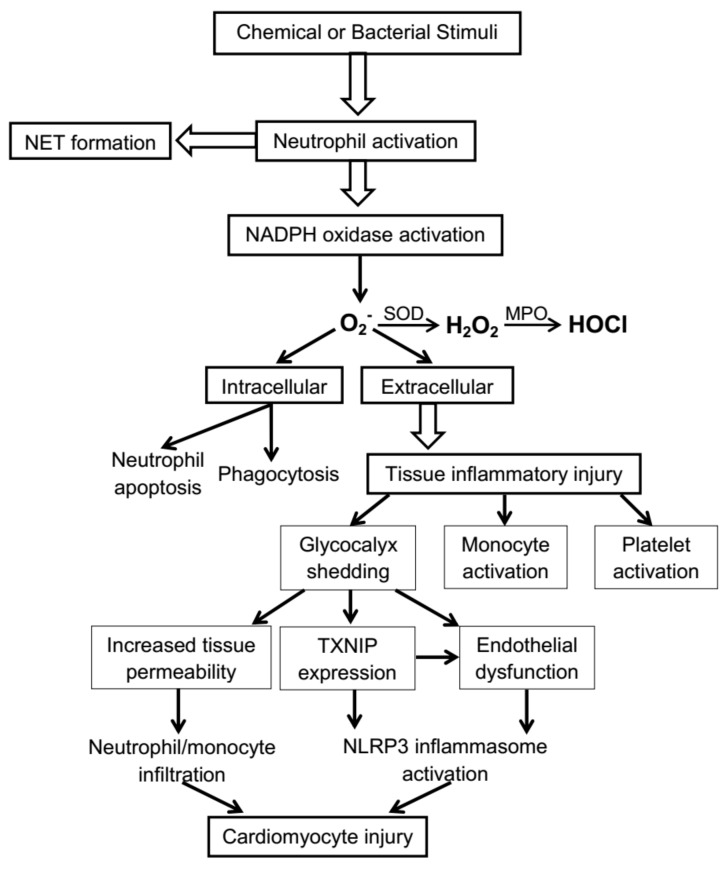
Cascade of intracellular and extracellular consequences of redox stress related to neutrophil activation. SOD = superoxide dismutase; MPO = myeloperoxidase; TXNIP = thioredoxin-interacting protein; NLRP3 = NOD-like receptor family, pyrin domain-containing 3.

One of the critically important aspects of the process of neutrophil activation is that a component of the resultant extracellular inflammatory functional disturbance induces the erosion of the vascular endothelial glycocalyx (a process known as glycocalyx shedding), largely via activation of a variety of matrix metalloproteinases [4]. Glycocalyx shedding, which can be detected and quantitated in vivo by assay of plasma concentrations of various glycocalyx components (e.g., sindecan-1), distorts vascular rheology, and results in the adhesion of neutrophils, monocytes and platelets to the damaged glycocalyx, as well as an increase in vascular permeability and potential infiltration of neutrophils and monocytes through the endothelium into the subjacent tissues—including both vascular smooth muscle and, in the case of the coronary vessels, the underlying myocardium [5]. Furthermore, distortion of vascular rheology from normal laminar flow patterns may result in dysfunction of the endothelium, with associated further ROS release, and increased tissue expression of the alpha-arrestin thioredoxin-interacting protein (TXNIP), which, inter alia, is an activator of the NLRP-3 (NOD-like receptor family, pyrin domain-containing 3) inflammasome [6]. Thus, a pro-inflammatory cascade may be amplified by neutrophil activation, with its final results within the heart including the development of inflammatory changes in both endothelial and myocardial cells, as shown in Figure 1.

Resolution of neutrophil-associated inflammation is also a fundamental aspect of the return to normal homeostasis: the “normal” “neutrophil burst” should be regarded as a transient response to a chemical irritant (not necessarily a bacterium—microcrystals, such as uric acid may also initiate the reaction). However, in practice, the resolution of tissue inflammation is sometimes slow, with a resultant increase in the probability of permanent tissue injury. In the case of the heart, this would involve apoptosis and necrosis of myocardial cells, and their eventual replacement by fibroblasts and myofibroblasts. Similarly, loss of glycocalyx from coronary arteries and arterioles may theoretically result in permanently disordered coronary vasomotor function. The clinical consequences may include not only impairment of the vasomotor reactivity of the coronary vascular bed, but also continued increases in vascular permeability, resulting in a propensity toward tissue oedema in the absence of the normal haemodynamic determinants of its development.

It is important to appreciate that persistence of an inflammatory response within neutrophil-infiltrated tissues represents a failure of normal neutrophil function. Many factors contribute to the normal “pro-resolution” role of previously activated neutrophils, including the expression of annexin-1 which promotes neutrophil apoptosis, anti-inflammatory chemokines such as interleukin 10, and also scavenging chemokines and cytokines [7]. Neutrophil-derived microparticles, released at the time of neutrophil activation, also play a part in the eventual reversal of this process [8]. Furthermore, several groups have found that the pro-inflammatory effects of interleukin-1 beta (mediated via increased ROS production, primarily from NADPH oxidase) are prolonged by *S*-glutathionylation of the p47phox component of the molecule [9].

The key point here remains the certainty that prolongation of leukocyte activation, irrespective of mechanism, together with excessive initial activation are likely to exacerbate tissue injury, whether in the myocardium, the vasculature or elsewhere. The remainder of this review will focus on potential ways of limiting inappropriately severe and/or prolonged neutrophil-initiated tissue inflammation, with particular reference to the role of BNP, before proceeding to briefly discuss some forms of disease affecting the myocardium where this pathophysiological interaction may represent a therapeutic opportunity.

### 1.2. Negative Modulation of Neutrophil Activation: The Role of cGMP Release

Neutrophils express nitric oxide synthases, which catalyse the generation of nitric oxide (NO) from arginine, leading to the activation of soluble guanylate cyclase (sGC) and the generation of cGMP, which is known to exert anti-inflammatory effects in general. However, it is also possible for excessive release of NO to induce tissue injury in a cGMP-independent manner: –NO may also combine with O_2_^−^, leading to the formation of peroxynitrite anion—which under conditions of rapid formation may induce nitrosative stress via the nitrosation of protein tyrosine residues—and activation of the “energy sink”/DNA reparative enzyme poly-(ADP/ribose) polymerase-1 (PARP-1)—which is implicated in the impairment of myocardial contractility and the potential induction of heart failure [10,11]. The available literature concerning the impact of exogenous NO administration on the course of neutrophil activation is relatively sparse. However, Klink et al. [12] have demonstrated a potentially protective effect, mediated largely or entirely by inhibition of phosphorylation of the NADPH oxidase subunit p47phox. On the other hand, there is evidence to suggest that NO may also accelerate this effect, potentially leading to sustained inflammatory activation [13]. These findings are consistent with the “Janus-type” effect of NO, perhaps dependent on the balance between sGC stimulation and the formation of peroxynitrite anion. Gross increases in NO release, such as are associated with amplified expression of inducible NO synthase in the presence of sepsis, might favour peroxynitrite-mediated adverse effects.

The other main source of potential activation of guanylate cyclase (but in this case particulate guanylate cyclase [pGC]) in neutrophils is BNP, which is increasingly released into the systemic circulation in a number of clinical disorders characterised by the presence of heart failure and/or inflammatory activation. We have recently made a number of important observations concerning interactions between BNP and neutrophil activation in a variety of heart failure syndromes [14,15]. These findings need to be set against a summary of the pathophysiology and biochemical effects of BNP release.

### 1.3. Synthesis and Storage of BNP

Although BNP was initially purified from porcine brain extracts [16] and given the name “brain natriuretic peptide”, the highest concentration of BNP is present in the heart [17]. As a primarily cardiac hormone, BNP is mainly synthesised in and secreted from the left ventricle [18]. The BNP gene encodes the prohormone proBNP. ProBNP is present in secretary granules within ventricular myocardium: it is released into extracellular space in response to various stimuli and then cleaved to BNP and the inactive N-terminal BNP fragment.

### 1.4. Release of BNP: Physiology and Pathology

In healthy subjects, mean BNP concentrations in venous blood are in the picomolar range. Although BNP has a half-life of only about 20 min [19], plasma BNP concentrations do not generally show rapid fluctuation. BNP secretion is largely controlled at the transcriptional level [20]. Prolonged stimuli generally increase both rates of synthesis and of secretion [21]. Intense exercise produces a moderate increase in plasma BNP concentration, and a greater exercise-related increase has been observed in individuals with left ventricular hypertrophy or heart failure [22].

Although stretch and variable left ventricular wall tension are likely to be important in controlling production and secretion of BNP, the precise mechanisms are still unclear. Increases in plasma BNP concentrations are seen in several pathological states, such as hypertrophic cardiomyopathy, dilated cardiomyopathy [23], and other forms of both systolic and diastolic heart failure [24]. Plasma BNP concentration is also elevated in pulmonary hypertension, probably reflecting secretion from the right ventricle [25]. Reduced oxygen tension has also been reported to stimulate BNP gene expression in cultured ventricular myocytes [26], and this may also be relevant in the contexts of both pulmonary hypertension and obstructive sleep apnoea. 

There is also evidence that increased release of BNP may occur in the presence of myocardial inflammation [27], despite minimal changes in cardiac distension. This will be discussed further in relation to the condition of takotsubo syndrome. 

Although the main source of circulating BNP is the heart, plasma levels of BNP can be affected by extra-cardiac disease states and also by factors that affect clearance of the peptide, such as variation in neutral endopeptidase (NEP) activity [28]. 

### 1.5. Physiological Actions of BNP

BNP acts through binding to natriuretic peptide receptor A (NPR-A, equivalent to particulate guanylate cyclase-A [pGC-A]) which results in the generation of the second messenger cGMP [29]. This intracellular cGMP is believed to produce cellular and physiological responses by interacting with cGMP-dependent protein kinases, cGMP-gated ion channels and cGMP-regulated cyclic nucleotide phosphodiesterases (PDEs) [30]. This means that cGMP regulates a number of intracellular processes, such as vascular smooth muscle relaxation [31], protection from oxidant damage [32], cellular proliferation [33], Ca^2+^ handling by the sarcoplasmic/endoplasmic reticulum ATPase [34] and the control of endothelial permeability [35]. Within the myocardium, it has recently been recognised that cGMP, in the presence of protein kinase G, promotes relaxation of the myocardium via phosphorylation of the sarcomeric titin molecule [36,37] which functions as a “giant spring.” Here, the resultant capacity for incremental myocardial relaxation during diastole may have profound implications on myocardial perfusion, particularly in the sub-endocardium, via reduction of extramural compression of coronary arteries during diastole.

On the other hand, BNP may function in parallel or in competition with NO, which also releases cGMP through the activation of sGC [38], and thus maintains cardiovascular and renal homeostasis [39]. In recent studies, evidence has accumulated regarding the cross-talk between these two enzymes (pGC-A and sGC) which represent “receptors” for BNP and NO, respectively [40]. 

In heart failure, BNP has numerous potentially beneficial effects, including diuretic, natriuretic, vasodilating [41], renin-angiotensin system suppressing [41], anti-fibrotic and anti-hypertrophic effects [41], as well as the inhibition of the synthesis of endothelin-1 [42]. In the presence of chronic heart failure, acute intravenous infusion of BNP improves central hemodynamics, including cardiac index, and also suppresses myocyte proliferation, cardiac growth, and compensatory cardiac hypertrophy [42].

### 1.6. Clearance of BNP

**Figure 2:**
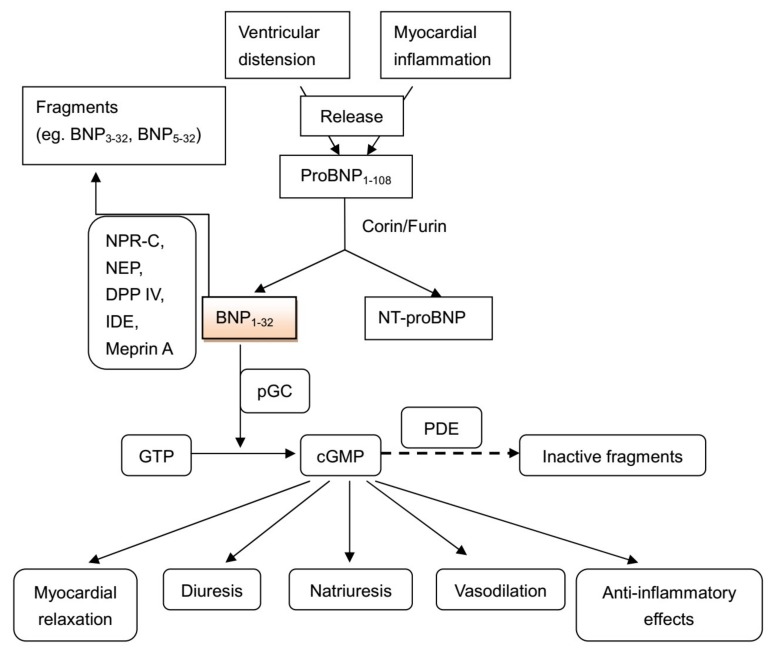
Cascade of BNP release and consequent physiological effects and clearance. BNP = B-type natriuretic peptide; NPR-C = natriuretic peptide receptor C; NEP = neutral endopeptidase; DPP IV = dipeptidyl peptidase IV; IDE = insulin degrading enzyme; pGC = particulate guanylate cyclase; PDE = phosphodiesterase; GTP = guanosine triphosphate; cGMP = cyclic guanosine monophosphate.

BNP is degraded through two well-characterised processes: (1) NPR-C-mediated internalization followed by lysosomal degradation; and (2) enzymatic degradation. It has been reported that the active form of BNP (BNP_1–32_) can be degraded by dipeptidyl peptidase IV (DPP IV), NEP, meprin and insulin degrading enzyme (IDE) to form BNP_3–32_, BNP_5–32_, BNP_8–32_ and smaller degradation peptides [43,44,45]. This cascade of events culminating in BNP release and associated vasodilatation is summarised in Figure 2.

### 1.7. Circulating BNP Fragments

While it is known that BNP represents an enzymatic cleavage product of the 108-amino acid precursor peptide proBNP, it has recently emerged that circulating BNP-like peptides include not only BNP, but also proBNP and a range of inactive cleavage products of proBNP [46]. This has been most intensely studied in acute heart failure, where most commercial BNP assay kits also detect several of the non-BNP peptides [46]. However, the extent to which this also occurs in normal subjects is less clear. 

Specifically, molecular analysis of “assayed BNP” in subjects with acute heart failure reveals two distinct peptides: a high-molecular weight form, proBNP_1–108_, and a low-molecular weight form, the biologically active BNP_1–32_ [47]. There is substantial cross-reactivity of the commercially available BNP and N-terminal pro B-type natriuretic peptide (NT-proBNP) assays with proBNP [46]. It appears that compared to BNP and NT-proBNP, proBNP_1–108_—the intact precursor peptide—circulates at high concentrations in patients with heart failure and may be the predominant form of circulating natriuretic peptide. In addition, several breakdown products of BNP_1–32_ circulate as well, and most of these degradation fragments of BNP_1–32_ are also detected by commercial BNP assays. Overall, it appears that there is relatively little bioactive BNP (BNP_1–32_) in the plasma of heart failure patients. These findings suggest there are abnormalities in the cleavage of proBNP_1–108_ to BNP_1–32_ in the presence of heart failure, but also do not permit evaluation of BNP_1–32_ concentrations within the myocardial interstitial space. 

## 2. BNP Suppression of Neutrophil ROS Formation: “BNP Resistance”

The concept that the anti-inflammatory effects of BNP might be related, in whole or part, to the suppression of ROS formation by neutrophils was previously investigated by our group [48]. We initially studied normal subjects to determine the effects of exogenous BNP on the “neutrophil burst” and associated myeloperoxidase (MPO) release. In this circumstance, BNP suppressed O_2_^−^ release, apparently in a cGMP/PKG-dependent manner (although no significant change in cGMP generation was detected). On the other hand, BNP did not affect MPO release. These data therefore suggested that under physiological conditions, BNP acts as a negative modulator of the “neutrophil burst.” The potential consequences of this interaction might include reduction in the extent of extracellular redox stress associated with neutrophil activation, and therefore the limitation of consequent tissue injury. The impact of exogenous BNP on the process of resolution of neutrophil activation was not explored.

**Figure 3:**
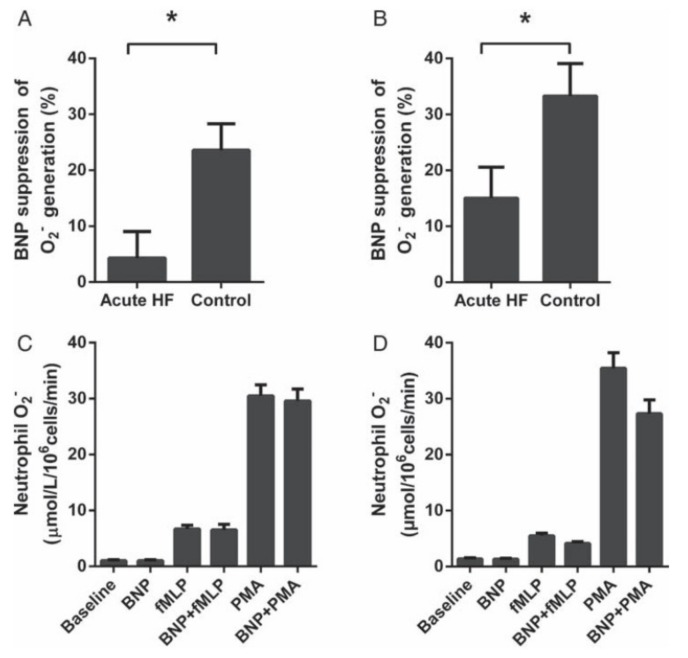
Comparison of BNP effects on phorbol 12-myristate 13-acetate (PMA)-stimulated (**A**) and *N*-formyl-methionyl-leucyl-phenylalanine (fMLP)-stimulated (**B**) O_2_^−^ generation by neutrophils from acute heart failure patients (*n* = 45) and control subjects (*n* = 29). (**C**,**D**) Raw data for actual O_2_^−^ production in neutrophils from acute heart failure patients (C) and control subjects (D). * *p* < 0.05 [14].

**Figure 4:**
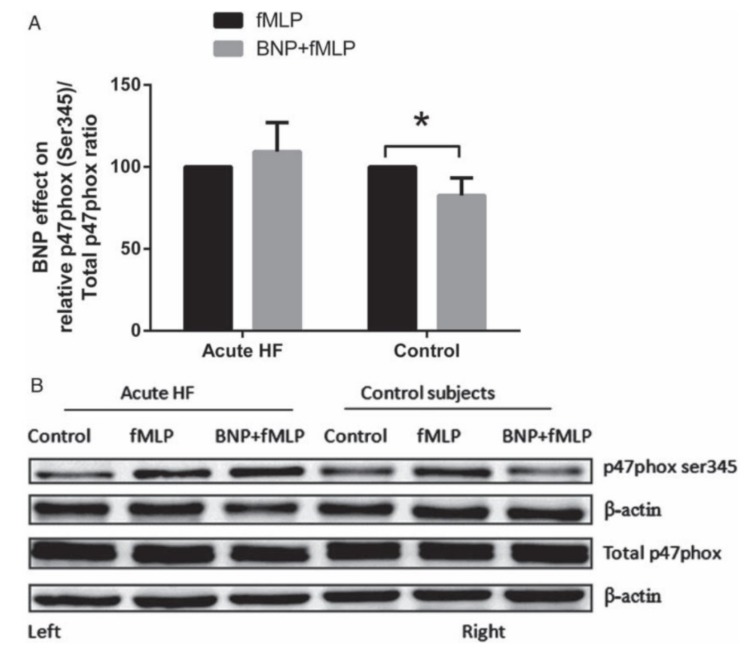
Effect of BNP on phosphorylation of p47phox Ser345. (**A**) In acute heart failure patients (*n* = 9) there is attenuation of BNP-induced suppression of p47phox Ser345 phosphorylation stimulated by *N*-formyl-methionyl-leucyl-phenylalanine (fMLP), which is seen in neutrophils from healthy subjects (*n* = 7). The relative ratio of p47phox Ser345 to total p47phox for samples treated with fMLP was taken as control (100%). * *p* < 0.05. (**B**) Representative immunoblots of acute heart failure patients and control subjects. Note suppression of phosphorylation of Ser345 by BNP in control subjects but not acute heart failure patients [14].

Subsequently, we sought to evaluate whether this “protective” effect of BNP might vary under conditions of acute myocardial inflammation, which particularly occur in patients with heart failure of recent onset. We compared the impact of exogenous BNP on the “neutrophil burst” in healthy subjects and in patients with acute heart failure. In the latter group, the impact of BNP in suppressing O_2_^−^ release was substantially diminished (Figure 3), and in association with this, it was found [14,48] that BNP inhibited phosphorylation of the NADPH oxidase subunit p47phox (and consequent activation of NADPH oxidase) only in normal subjects (Figure 4). These observations therefore implied that acute heart failure is associated with impairment of the anti-oxidant effects of BNP at the neutrophil level, leading to net increases in the formation of O_2_^−^ and its potential extracellular release, and thus rendering patients more prone to tissue injury. Interestingly, this “BNP resistance” partially resolved over a mean period of five weeks of anti-failure therapy. Furthermore, while we did not explore whether greater infiltration of myocardium with leukocytes in heart failure might reflect poor tissue responsiveness to BNP, this has been suggested by previous investigations by Kawakami et al. [49].

## 3. Is BNP Synergistic with NO?

Unfortunately, the potential existence of interactions between the NO/sGC and the BNP/pGC systems has received little attention to date. Theoretically, NO, which also inhibits NADPH oxidase assembly [50] might augment the effects of BNP in limiting the “neutrophil burst” and the associated release of ROS. On the other hand, NO may also initiate sGC-independent pathophysiological effects, such as extensive protein nitrosation and activation of the “energy sink” enzyme PARP-1 [11]. Clearly this area merits further investigation. Furthermore, expression of the inducible form of NOS (iNOS), leading to massive increases in NO release, may represent a special case which may be of relevance to patients with sepsis. 

## 4. Potential Clinical Implications

The abovementioned findings have a number of clinically relevant therapeutic implications. It is clear that BNP acts as a substantial negative modulator of O_2_^−^ release by neutrophils, and lacks the potential (peroxynitrite-mediated) negative implications of marked release of NO within the myocardium. Therefore, BNP would tend to limit tissue injury associated with excessive activation of NADPH oxidase. 

There are a number of acute and chronic cardiac disorders which are characterised by the presence of myocardial infiltration by neutrophils, with associated tissue inflammation, and with both the marked release of BNP and the activation of glycocalyx shedding—a result of the impact of a range of matrix metalloproteinases [5]. These include, in particular, acute myocardial infarction [51] and takotsubo syndrome [52], a catecholamine-initiated form of acute myocardial inflammation. In both of these conditions, myocardial infiltration by neutrophils and monocyte/macrophages is prominent. In patients with acute heart failure, including those without significant myocardial ischaemia, BNP release is marked, and this remains the case to a latter extent in chronic heart failure, where persistence of myocardial inflammation is also a frequent finding [53]. 

Indeed, in takotsubo syndrome, BNP release occurs to extraordinary extents, especially compared with the usually minor elevation of left ventricular filling pressures. These findings suggest that in this condition in particular, BNP may be released mainly in response to the beginning of intra-myocardial inflammation, which is particularly intense in the early stages of takotsubo syndrome. On the other hand, it is likely that excessive NO release and effects—including the induction of nitrosative stress [54]—are also relevant to the development of myocardial inflammation in such patients. A major problem in the early stages of takotsubo syndrome is the development of hypotension and shock, and it is certainly possible that effective infusion of BNP, rather than of NO, might provide a means for limiting the extent of myocardial inflammation and the resultant prolonged impairment of left ventricular function.

Counter-intuitively, infusion of the BNP analogue nesiritide has been found to be of little use in the treatment of acute heart failure [55]. This lack of effect implies tissue resistance to the biochemical and/or physiological effects of BNP in this circumstance, consistent with our observations regarding the interactions of BNP with the “neutrophil burst” [14]. However, it is surprising that therapeutic response to nesiritide remains poor despite relatively high infusion rates.

Importantly, it is not yet clear whether impairment of responses to BNP, other than inhibition of the “neutrophil burst,” occurs in either heart failure (acute/chronic; systolic/diastolic) or takotsubo syndrome. Specifically, it would be of advantage to know to what extent the vasodilator and anti-inflammatory effects of BNP might be compromised in these conditions. 

The issue may be particularly important in heart failure with a preserved ejection fraction, a very common condition in ageing individuals, and one in which it has been postulated that ongoing systemic inflammatory activation may be critical to the development and perpetuation of heart failure [37]. It must be noted that, thus far, the medical literature on the limitation of chronic myocardial and coronary microvascular inflammation is fragmentary, but progress in quantitation of myocardial inflammation and resultant oedema utilizing cardiac magnetic resonance imaging should facilitate the extension of clinically-based investigations in this area.

These findings have a number of potential therapeutic sequelae. It seems likely, but not yet certain, that impairment of tissue responsiveness to BNP may result from the impact of oxidative stress on activity of pGC. Hence, two potential therapeutic avenues are theoretically available to limit myocardial and coronary vascular endothelial injury induced by “BNP resistance.” First, therapy with agents known to limit activation of NADPH oxidase, such as ACE inhibitors and perhexiline [56] has already been shown to be useful, although the contribution of BNP potentiation to salutary effects at the level of clinical end-points has not yet been explored. Second, there is evidence that degradation of BNP by NEP within neutrophils may limit its effectiveness [57]. Hence, the increasing clinical use of therapy, including NEP inhibitors, in the management of chronic heart failure [58] may be regarded as a potential first step toward the additional utilization of such therapy in the treatment of acute cardiac emergencies.

## 5. Conclusions

The “neutrophil burst” represents a pivotal component of innate immune defense against invading micro-organisms, but also a cause of “collateral tissue damage” via extracellular effects of the O_2_^−^ anion radical. In the heart, release of O_2_^−^ induces tissue injury (both myocardial and microvascular), as is seen in many forms of acute heart failure. Takotsubo syndrome provides a particularly apposite example of this cascade of events. Moreover, failure of resolution of neutrophil activation can lead to progressive worsening of tissue damage. BNP appears to function physiologically as a negative modulator of the “neutrophil burst,” which thus may limit resultant tissue injury. However, this protective effect is lost during the acute phase of heart failure. We propose that optimizing the efficacy of BNP as a counterbalance against neutrophil activation represents a theoretically promising therapeutic strategy to treat or prevent heart failure.

## Abbreviations

BNP	B-type natriuretic peptide
DPP IV	Dipeptidyl peptidase IV
fMLP	*N*-formyl-methionyl-leucyl-phenylalanine
HOCl	Hypochlorous acid
IDE	Insulin degrading enzyme
MPO	Myeloperoxidase
NEP	Neutral endopeptidase
NET	Neutrophil extracellular trap
NLRP3	NOD-like receptor family, pyrin domain-containing 3
NPR-A	Natriuretic peptide receptor A
NPR-C	Natriuretic peptide receptor C
NO	Nitric oxide
O_2_^−^	Superoxide anion radical
PARP-1	poly-(ADP/ribose) polymerase-1
PDE	Phosphodiesterase
pGC	Particulate guanylate cyclase
PMA	Phorbol 12-myristate 13-acetate
ROS	Reactive oxygen species
sGC	Soluble guanylate cyclase
SOD	Superoxide dismutase
TXNIP	Thioredoxin-interacting protein
